# Perfringolysin O Theta Toxin as a Tool to Monitor the Distribution and Inhomogeneity of Cholesterol in Cellular Membranes

**DOI:** 10.3390/toxins8030067

**Published:** 2016-03-08

**Authors:** Masashi Maekawa, Yanbo Yang, Gregory D. Fairn

**Affiliations:** 1 Keenan Research Centre for Biomedical Science, St. Michael′s Hospital, 209 Victoria Street, 6th Floor, Toronto, ON M5S 1T8, Canada; yanbo.yang@mail.utoronto.ca; 2 Department of Biochemistry, University of Toronto, Toronto, ON M5S 1A8, Canada; 3 Department of Surgery, University of Toronto, Toronto, ON M5T 1P5, Canada; 4 Institute of Medical Science, Faculty of Medicine, University of Toronto, Toronto, ON M5S 1A8, Canada; 5 Institute for Biomedical Engineering and Science Technology (IBEST), Ryerson University and St. Michael′s Hospital, Toronto, ON M5B 2K3, Canada

**Keywords:** cholesterol, perfringolysin O, biosensor, membranes, microscopy

## Abstract

Cholesterol is an essential structural component of cellular membranes in eukaryotes. Cholesterol in the exofacial leaflet of the plasma membrane is thought to form membrane nanodomains with sphingolipids and specific proteins. Additionally, cholesterol is found in the intracellular membranes of endosomes and has crucial functions in membrane trafficking. Furthermore, cellular cholesterol homeostasis and regulation of *de novo* synthesis rely on transport via both vesicular and non-vesicular pathways. Thus, the ability to visualize and detect intracellular cholesterol, especially in the plasma membrane, is critical to understanding the complex biology associated with cholesterol and the nanodomains. Perfringolysin O (PFO) theta toxin is one of the toxins secreted by the anaerobic bacteria *Clostridium perfringens* and this toxin forms pores in the plasma membrane that causes cell lysis. It is well understood that PFO recognizes and binds to cholesterol in the exofacial leaflets of the plasma membrane, and domain 4 of PFO (D4) is sufficient for the binding of cholesterol. Recent studies have taken advantage of this high-affinity cholesterol-binding domain to create a variety of cholesterol biosensors by using a non-toxic PFO or the D4 in isolation. This review highlights the characteristics and usefulness of, and the principal findings related to, these PFO-derived cholesterol biosensors.

## 1. Introduction

Most of the cholesterol-dependent cytolysins (CDCs) are bacterial pore-forming toxins that specifically bind to cholesterol in the plasma membrane of mammalian cells [1,2,3]. Intermedilysin is an exception to the cholesterol requirement as intermedilysin makes use of CD59 for binding to the plasma membrane [4]. CDCs are secreted by a variety of Gram-positive bacteria including but not limited to *Bacillus*, *Listeria*, *Streptococcus*, *Clostridium*, and *Lactobacillus* [5]. Binding of these water-soluble CDCs to cholesterol in the exofacial leaflets of the plasma membrane induces the oligomerization of the toxins (30–50 monomers) [1,6,7,8]. Through this oligomerization, CDCs form pores (~300 Å in a diameter) in the plasma membrane by inserting their large β-barrel domains, leading to cell lysis [9,10,11,12].

Perfringolysin O (PFO), secreted by *Clostridium perfringens,* has been a prototype of CDCs and as such has been extensively studied [13,14,15,16,17,18,19,20]. The ability of PFO to recognize cholesterol has motivated researchers to develop PFO-derived cholesterol biosensors similar to the use of other toxins to visualize lipids. For examples, the non-toxic fragments of the lysenin and equinatoxin II are excellent biosensors of sphingomyelin [21,22,23,24]. Another example of toxins used as tools for cell biology include the non-toxic B subunits of Cholera and Shiga toxins (CTxB and STxB, respectively) that bind to the exofacial glycosphingolipids GM1 and Gb3, respectively [25,26]. Thanks to both the development of these lipid biosensors and advances in microscopic techniques, our understanding of cell biology continues to progress, especially in the field of lipid trafficking. From this standpoint, PFO has been an attractive toxin for the design of cholesterol biosensors.

Cellular membranes are formed by a bilayer of phospholipids with cholesterol and sphingolipids [27,28]. Cholesterol is enriched in the plasma membrane of animal cells, with nearly half of the lipid molecules being cholesterol [29]. Lipid researchers had believed for a long time and actually shown that acyl chains of phospholipids can interact with the four fused rigid rings of cholesterol through van der Waals interactions for tight packing of cholesterol in the model membranes (the condensed complex model) [30,31,32]. In addition, in cellular membranes, the small hydroxyl group of cholesterol is not sufficient to shield its relatively large hydrophobic structure from water. Thus, cholesterol is thought to be closely associated with phospholipids containing a comparatively large hydrophilic head group and long saturated fatty acyl chains such as sphingomyelin (the umbrella model [28]). Together, the umbrella model and biochemical experiments have led to the establishment of the notion that cholesterol and sphingolipids form nanodomains with particular proteins in the exofacial leaflets of the plasma membrane, typically referred to as lipid rafts [33]. Cholesterol in these lipid rafts and other nanodomains of the plasma membrane has crucial roles in cell signaling, endocytosis, viral infection, and Alzheimer′s disease [34,35,36]. In addition to the functions of cholesterol in the plasma membrane, cholesterol has important roles in vesicular trafficking, especially in the recycling pathways [37,38,39,40,41]. Accordingly, cholesterol is enriched in the transferrin receptor (TfR)-positive recycling endosomes [42,43].

Cellular cholesterol is derived from either the *de novo* synthesis pathway or by the uptake of lipoproteins, especially low-density lipoprotein (LDL). After the internalization of LDL via the LDL receptor, LDL is transported to the multivesicular bodies (MVBs) through the vesicular pathway and subsequently degraded releasing cholesterol [28]. Non-esterified cholesterol generated from LDL in the late endosomes utilizes two proteins, Niemann-Pick Type C1 (NPC1) and C2 (NPC2), to move out of the endosomal systems, although the precise mechanism remains unclear [28]. However, both vesicular and non-vesicular transport contribute to the proper distribution of cholesterol and other lipids [28,44,45,46,47].

Comprehensive understanding of the cellular functions and characteristics of cholesterol in greater detail necessitates visualization of the subcellular distribution of cholesterol. To achieve this purpose, two canonical cholesterol probes have been established, namely filipin and fluorophore conjugated-cholesterol [48]. Filipin is an intrinsically fluorescent molecule and can bind to cholesterol directly [49]. However, filipin is a UV-excitable dye and can be photo-bleached, making it difficult to obtain high-quality images [48]. The other type of cholesterol probe commonly used is cholesterol directly labeled with a fluorophore (e.g., Bodipy, TopFluor) and subsequently introduced into the cell [50,51]. Although these fluorophores are stable and easy to observe, one limitation is that the addition of the comparatively large fluorophore alters the biophysical properties of cholesterol [52]. Furthermore, both filipin and the fluorophore-labeled cholesterol cannot distinguish the transbilayer distribution of cholesterol [48].

**Figure 1:**
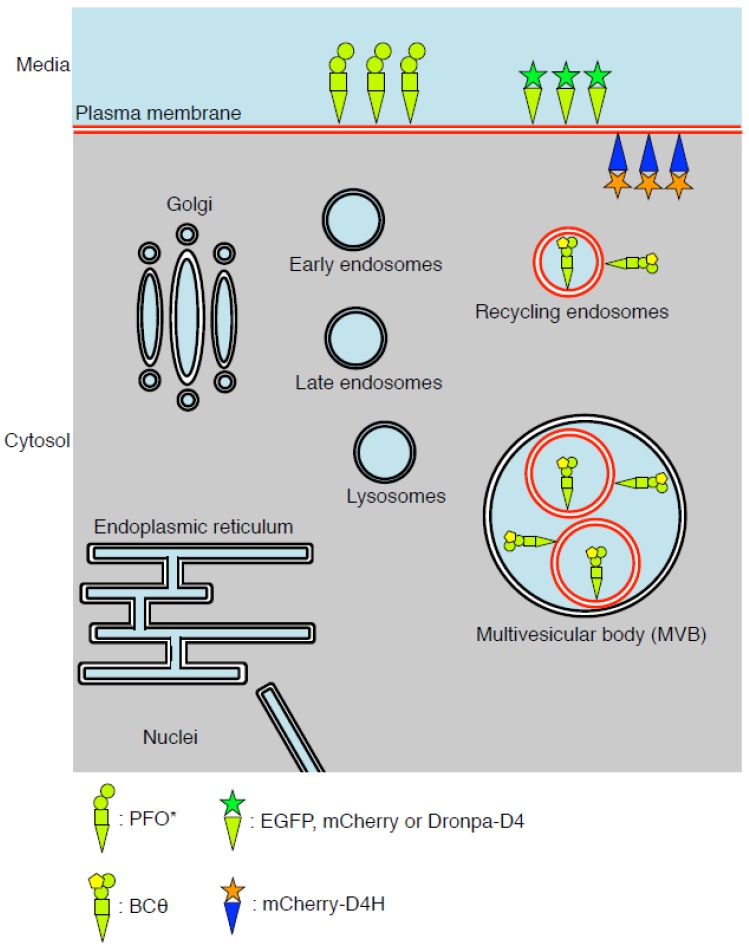
Cholesterol-rich membranes visualized by the Perfringolysin O (PFO)-derived cholesterol biosensors. Cholesterol-rich membranes (red lines) detected or visualized by the PFO-derived cholesterol biosensors are shown. Radioisotope-labeled PFO ^(Y181A/C459A)^ also refered to as PFO* detects cholesterol in the exofacial leaflets of the plasma membrane by incubation at 4 °C. Recombinant fluorophore labled-D4 proteins can visualize cholesterol in the exofacial leaflets of the plasma membrane by incubation at room temperature. BCθ are accumulated in the internal membranes of the multivesicular bodies (MVBs) and recycling compartments in the fixed cells. Cytosolic mCherry-D4H localizes in the inner leaflet of the plasma membrane.

To overcome the limitations of filipin and the fluorophore-labeled cholesterol, biosensors have been developed by making use of the non-toxic full length of PFO or domain 4 of the toxin (D4) (see Table 1). Researchers have made use of different strategies to make PFO-derived proteins useful as probes or sensors including introducing radioisotopes, biotin, or fluorophores. Subsequent studies have identified cholesterol-rich membranes in the cell using these PFO-derived cholesterol biosensors with the suitable detectors and microscopes (Table 1 and Figure 1). In this review, we highlight the usefulness of each PFO-derived cholesterol biosensors, and we also show the major findings provided by those cholesterol biosensors.

## 2. Structure and Mutations of PFO

**Figure 2:**
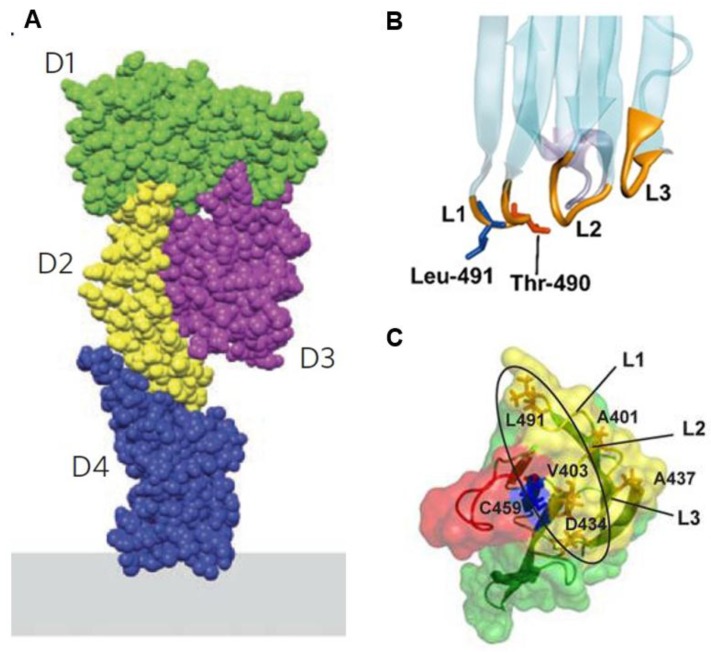
Domain structure of PFO. (**A**) The PFO monomer with the four domains is indicated in orientation to the membrane. Domain 4 (D4) directly associates to the membrane through the recognition of cholesterol. The scheme is reproduced from [12], Copyright Nature Publishing Group, 2013. (**B**) The location of the two amino acid residues (Threonine-490 and Leucine-491) in D4 required for cholesterol binding are shown. Loops L1–L3 at the base of D4 insert into the membrane in the cholesterol-dependent manner. The scheme is reproduced from [57] Copyright PNAS, 2010. (**C**) The bottom view of D4 is shown. Substitution of Aspartic acid-434 to Serine increases the affinity of PFO to cholesterol. The schematic is reproduced from [20], Copyright American Chemical Society, 2012.

The three-dimensional determination of the PFO reveals a monomer consisting of four distinct domains (Figure 2A; [16]). Domain 2 (D2) has an elongated structure that links to Domain 1 (D1) for connecting Domain 3 (D3) and Domain 4 (D4) (Figure 2A; [16]). D1 is comprised of a seven-stranded antiparallel β-sheet and four α-helixes [16]. D2 contains one β-sheet consisting of four mixed β-strands [16]. D3 consists of one α-helix, two sets of three α-helixes, and one five-stranded antiparallel β-sheet, of which the three α-helices and β-sheet construct a three layer structure of α/β/α [16]. D4 contains one four-stranded antiparallel β-sheet and one four-stranded β-sheet with a mixed topology that are orientated into a β-sandwich structure connected by four loops [11,16]. The cytolysis of eukaryotic cells by PFO begins by D4-mediated binding to the plasmalemmal cholesterol [11,18,53,54]. D4 interacts with the membrane only through the tip of the four connecting loops, while the rest of D4 has no contact with the membrane [11,54]. Importantly, D4 was shown to be sufficient for PFO to bind to cholesterol in the plasma membrane [55]. After PFO binds to the plasma membrane through the recognition of cholesterol by D4, PFO forms oligomers containing 35–50 monomers [1,6,7,8]. This oligomerization induces vertical collapse of D2 and allows two amphipathic transmembrane domains of D3 to cross the plasma membrane, leading to pore formation and cell lysis; the D1 domain functions to orchestrate structural transitions of other domains within the process of pore formation [1,56].

**Figure 3:**
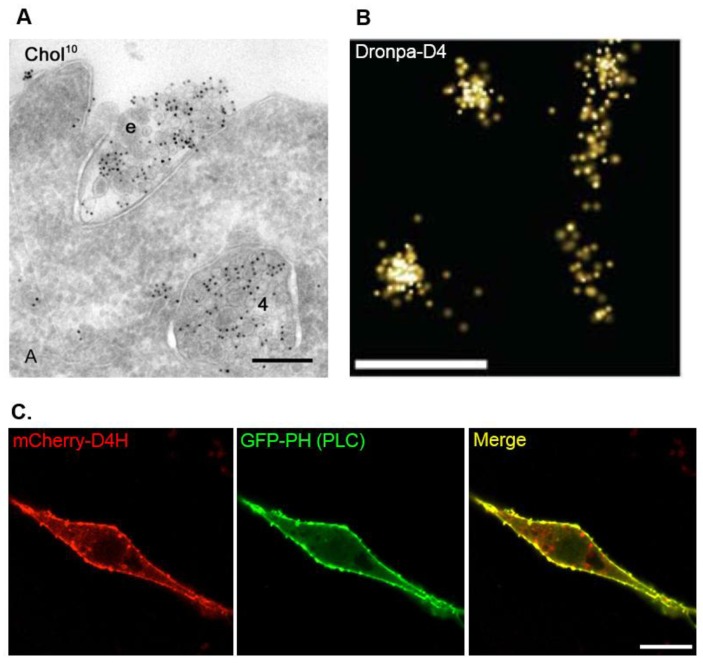
Examples of images visualized by the PFO-derived cholesterol biosensors. (**A**) Cholesterol-rich organelles in the human B-lymphocytes cells were visualized by BCθ using immunoelectron microscopy. (e; exosomes, 4; multivesicular bodies (MVBs)). Bar, 200 nm. The image is reproduced from [62] Copyright John Wiley & Sons, 2003. (**B**) Recombinant Dronpa-D4 protein visualized by photoactivated localization microscopy (PALM) reveals cholesterol clusters in the exofacial leaflets of the plasma membrane in HeLa cells. Bar, 500 nm. The image is reproduced with permission from [63], Copyright Royal Society of Chemistry, 2011. (**C**) Cholesterol in the cytosolic leaflets of the plasma membrane is visualized by expression of mCherry-D4H in the cytosol of Raw264.7 cells. GFP-PH (PLC) is used as a plasma membrane marker. Bar, 10 μm.

To date, a variety of mutations in PFO that affect cholesterol-binding or pore formation has been reported. One of the four loops in domain 4 is the conserved undecapeptide that is rich in tryptophan [58]. Replacement of the tryptophan residues with phenylalanine in the undecapeptide impairs the cytotoxic activity [59]. This is consistent with the observations that the undecapeptide is involved in binding of PFO to the membrane and the subsequent conformational changes, which ultimately lead to the insertion of the β-barrel in the plasma membrane [58,59,60]. Additionally, evidence showed that the modification of cysteine in the undecapeptide would inhibit the transition from pre-pore to pore [58]. In the tip of loop L1 of D4, a threonine–leucine (T490–L491) pair is critical for the specific recognition and binding of PFO to membrane cholesterol (Figure 2B; [57]). This Thr-Leu motif is also known as the cholesterol recognition/binding motif (CRM), which has been shown to be conserved in all identified cholesterol-dependent cytolysins [57]. The structural arrangement of CRM is relatively inflexible [57]. Hence, any mutations such as conservative substitution, and inverting their relative positions (PFO T490L/L491T), results in a dramatic decrease in the ability to bind to cholesterol [57]. Additional studies have identified amino acid residues in D4 that change the threshold for cholesterol binding of PFO [20,61]. For instance, substitution of the cysteine at 459 in the undecapeptide to the alanine (C459A; Figure 3C) increased the threshold for cholesterol-binding of PFO from 30 to 35 mol % [61]. On the other hand, substitution of the negatively charged aspartic acid at 434 located in the loop L3 in D4 for the serine (D434S; Figure 3C) decreased the threshold for cholesterol-binding of PFO [20]. Thus, when we chose to generate a plasmid-based biosensor we used the D434S mutant [43].

## 3. PFO*, a Non-Toxic PFO, for Detection of Cholesterol in the Exofacial Leaflets of the Plasma Membrane

Previous studies have shown that the Y181A mutation of PFO causes it to lose its pore formation ability, although the mutant is still able to bind to cholesterol [17,19]. By making use of this mutation, a non-toxic PFO, called PFO*, was developed [64]. The Y181A mutation was introduced into the cysteine-less (C459A), fully-active PFO, with the resulting double mutant termed PFO*. Both PFO and PFO* bound to the liposomes in which cholesterol concentration was more than 40 mol % [64]. PFO* formed pores in the plasma membrane of the mammalian cells at 37 °C as normal PFO does; however, incubation of cells with PFO* at 4 °C did not induce the permeabilization of the plasma membrane [64]. Normal PFO still induced permeabilization of the plasma membrane by pore formation at 4 °C [64]. This property of PFO* enabled us to detect cholesterol in the exofacial leaflets of the plasma membrane by treatment of cells with radioisotope labeled PFO* (^125^I-labeled PFO*) at 4 °C (Table 1). Binding of ^125^I-labeled PFO* to the exofacial leaflets of the plasma membrane was abolished in the cholesterol-depleted cells [64]. Also, ^125^I-labeled PFO* is useful for detecting the LDL-derived cholesterol transport from a lysosome to the plasma membrane [64]. By incubating cholesterol-depleted cells with LDL, binding of ^125^I-labeled PFO* to the exofacial leaflets of the plasma membrane increased progressively [64]. Importantly, in the presence of chloroquine, which inhibits the degradation of LDL in lysosomes, ^125^I-labeled PFO* binding to LDL-loaded, cholesterol-depleted cells was not observed [64], confirming that cholesterol derived from exogenous LDL is transported to the plasma membrane after degradation in lysosomes. Attention should be paid for this biosensor because PFO* can detect only cholesterol in the exofacial leaflets of the plasma membrane. Thus, it was impossible to detect cholesterol trafficking in the organelles between lysosomes and the plasma membrane.

The use of ^125^I-labeled PFO* demonstrated the existence of three discrete “pools” of plasmalemmal cholesterol [65]. These pools were classified as (a) PFO-accessible cholesterol; (b) sphingomyelin (SM)-sequestered cholesterol; and (c) essential cholesterol. By treatment of cells with sphingomyelinase (SMase) that hydrolyzes SM, binding of ^125^I-labeled PFO* to the exofacial leaflets of the plasma membrane increased due to the liberation of the previously SM-sequestered pool. This SM-sequestered cholesterol exhibited additional characteristics. Finally, the authors defined a third pool of cholesterol in the exofacial leaflets of the plasma membrane as essential cholesterol, although the biological meanings of this essential cholesterol in the plasma membrane remain unclear. Further characterization of these three cholesterol pools would be a topic for future work.

## 4. BCθ, a Biotinylated PFO Fragment for Visualization of Cholesterol in the Endocytic Pathway

To examine the distribution of cholesterol in the luminal leaflets of the endocytic pathways, previous studies made use of a biotinylated fragment of PFO generated by incubation with subtilisin Carlsberg and immunolabeling electron microscopy [66,67]. The biotinylated PFO was generated stepwise. First, PFO was protease-nicked to inactivate cell lysis activity and this modified PFO was called Cθ [68]. Next, Cθ was methylated to generate MCθ and then biotin was conjugated to MCθ [69]. Importantly, the affinities to cholesterol of those modified PFO are similar to normal PFO and have no cytotoxicity [14,68,69,70]. Ultrathin cryosections of the human lymphoblastoid cells were prepared after fixation with formaldehyde [67]. Next, the sections were incubated with biotinylated PFO theta toxin, called BCθ ([66]; Table 1), and fixed with glutaraldehyde. Subsequently, BCθ was detected with the anti-biotin antibody and protein gold A or Avidin-gold conjugate. This immunoelectron microscopy provides images with high resolution (15–30 nm) and facilitates the visualization of intracellular structures and molecules in superior detail. The method clearly identified the plasma membrane, intraluminal vesicles within multivesicular bodies (MVBs) and exosomes at the cell surface as cholesterol-rich organelles (Figure 3A; [62,67]). In contrast, the limiting membrane of the MVBs and aspects of the Golgi were largely negative. These observations are reasonable as those cholesterol-rich internal membranes within the MVBs could be secreted as exosomes by fusion of the MVBs with the plasma membranes [62]. Additionally, BCθ was also detected in the transferrin receptor (TfR) or cation-dependent mannose-6-phosphate receptor positive tubular vesicles [62], showing that cholesterol is also rich in the recycling compartments. Consistent with this finding, a previous study has shown that cholesterol is enriched in the TfR-positive endosomal fractions of MDCK cells [42]. Although immunoelectron microscopy does not allow live imaging, a combination of BCθ and immunoelectron microscopy can provide useful information about subcellular cholesterol distribution with the highest resolution.

## 5. D4 for the Visualization of Cholesterol Domains in the Exofacial Leaflets of the Plasma Membrane

The results of biochemical experiments have led to the hypothesis that cholesterol, sphingomyelin, and specific proteins form domains in the exofacial leaflets of the plasma membrane typically referred to as lipid rafts [33]. Lipid rafts are thought to be important for various cellular functions such as cell signaling, endocytosis, and viral infections [34,35,36]. In this lipid rafts hypothesis, researchers propose that cholesterol is distributed as “clusters” in the lipid rafts at the exofacial leaflets of the plasma membrane. Recently, the use of stochastic superresolution microscopy has successfully been used to visualize cholesterol clusters in the exofacial leaflets of the plasma membrane using the recombinant D4 proteins (Table 1; [63]). By a combination of the Dronpa tagged recombinant D4 proteins and photoactivation localization microscopy (PALM), two types of cholesterol clusters in the exofacial leaflets of the plasma membrane were visualized in HeLa cells with high resolution (Figure 3B; [63]). The first type of cholesterol clusters was observed as lines with widths of approximately 150 nm and lengths of approximately 0.7 to 5.5 μm (Figure 3B; [63]). Mizuno *et al.* concluded that these elongated cholesterol clusters were microvilli, consistent with their abundance of the dorsal side of HeLa cells and their extensive lipid rafts [63,71,72]. The other type of cholesterol clusters in the exofacial leaflets of the plasma membrane showed round shapes with an average radius of 118 nm calculated by Ripley′s K-function analysis (Figure 3B; [63,73]). These observations strongly support the hypothesis that cholesterol forms nanodomains in the exofacial leaflets of the plasma membranes. Furthermore, a recombinant mCherry-D4 protein was used to visualize cholesterol-rich domains in the exofacial leaflets of the plasma membrane of the red blood cells by wide-field microscopy [74]. These cholesterol-rich domains in the exofacial leaflets of the plasma membrane overlapped extensively with BODIPY-sphingomyelin and these domains were sphingomyelin-dependent, temperature-sensitive, immobile, and spectrin-dependent [74]. However, at this point, the relevance of these domains in red blood cells and their actual dimensions are unclear.

Visualization of the cholesterol-rich domains in the exofacial leaflet of the plasma membrane using conventional fluorescence or super-resolution methods will enable researchers to investigate the regulatory mechanisms of their formation. For example, researchers can investigate the contribution of the cytoskeleton (e.g., actin, microtubules) and the membrane phospholipids (e.g., sphingomyelin, phosphatidylserine) to the formation of these cholesterol-rich domains. Further examination of whether the cholesterol clusters exist not only in the exofacial leaflets but also in the cytosolic leaflets of the plasma membrane would be important future work (see also Section 6).

## 6. D4H for Visualization of Cholesterol in the Cytosolic Leaflets of the Cellular Membranes

Visualization of cholesterol in the cytosolic leaflet of the plasma membrane has been challenging because of the lack of suitable biosensors [48]. Filipin, which can bind cholesterol specifically [49], can visualize the cellular cholesterol; however, filipin cannot distinguish cholesterol in the cytosolic leaflet from that in the luminal (or exofacial) leaflets of the cellular membranes. Furthermore, filipin is not available for live imaging because of its membrane permeability activity [75,76]. Fluorophore-labeled cholesterols such as BODIPY-cholesterol and TopFluor-cholesterol [50,51] are available for live imaging. However, these dyes do not have the same biophysical properties of cholesterol in the cellular membranes. To overcome these issues, we recently established a new vector-based biosensor of cholesterol called D4H. When expressed in the cytosol the mCherry-D4H fusion protein binds to cholesterol in the cytosolic leaflets of the cellular membranes (Table 1 and Figure 3C; [43]). In the initial experiments, we found that mCherry-tagged D4 expressed in cytosol did not localize to the plasma membrane unless excess cholesterol was loaded using MβCD [43]. Thus, we speculated that the affinity of the wild-type D4 to cholesterol was not sufficient to recognize cholesterol in the cytosolic leaflets of the plasma membrane. A previous study has identified point mutations that change the affinity of PFO to cholesterol [20]. Among those mutations, we focused on the D434S mutation in D4, which increases the affinity to cholesterol (Figure 2C; [20]). We expected that this mutant construct with higher affinity to cholesterol could localize to the cytosolic leaflets of the plasma membrane without loading the exogenous cholesterol. As expected, the cytosolically expressed mutant construct of D4 (D434S), called D4H, localized in the cytosolic leaflets of the plasma membrane in a cholesterol-dependent manner [43].

Using this new cholesterol biosensor for the cytosolic cholesterol in the cellular membranes, we also showed that phosphatidylserine (PtdSer), one of the major phospholipids in the cytosolic leaflet of the plasma membrane, is essential for retaining of cholesterol in the inner leaflet [43]. In the PtdSer manipulated cells [77,78], in which the lipid is depleted from the plasma membrane, mCherry-D4H was dissociated from the cytosolic leaflets of the plasma membrane [43]. Interestingly, in those cells, binding of the EGFP-D4 recombinant proteins to the exofacial leaflets of the plasma membrane was increased [43]. These observations are consistent with the notion that cholesterol redistributes to the exofacial leaflet of the plasma membrane upon the depletion of PtdSer. Importantly, supplementation of the exogenous PtdSer restored the defects of transbilayer distribution of cholesterol in the plasma membrane [43]. Furthermore, we found that 1-stearoyl-2-oleoyl PtdSer (SOPS) can specifically and physically interact with cholesterol in the model membranes [43]. Collectively, our study suggested a new concept: that PtdSer retains cholesterol in the cytosolic leaflets of the plasma membrane through physical interaction. A recent publication reported that PtdSer is essential for the nano-clustering of GPI-anchored proteins in the exofacial leaflets of the plasma membrane [79]. PtdSer-dependent cholesterol transbilayer distribution might explain the PtdSer-dependent, GPI-anchored protein clusters.

Recently, the D4H probe was used for high-resolution immunoEM. Also, very recently, observation of the D4H and Lact-C2, a PtdSer biosensor [80], in the cytosolic leaflets of the plasma membrane in high-resolution was performed with by electron microscopy [81]. Cho *et al.* found that treatment of MDCK cells with mβCD that acutely extracts cholesterol from the plasma membrane induced the internalization of PtdSer from the plasma membrane into the endosomes [81]. In this condition, mβCD treatment disrupted the spatial organization of both cholesterol and PtdSer that remained in the cytosolic leaflets of the plasma membrane [81]. In that study, Cho *et al.* identified another drug, fendline (an acid sphingomyelinase inhibitor; [82,83]), which caused the internalization of both cholesterol and PtdSer from the plasma membrane into the intracellular compartments [81]. Very interestingly, fendline treatment disrupted the spatial organization of PtdSer [81]. These data reveal the existence of the different PtdSer-nanodomains in the cytosolic leaflets of the plasma membrane, some of which are cholesterol dependent while others appear cholesterol independent [81,84]. Collectively, D4H is a useful probe to analyze the moiety of cholesterol in the cytosolic leaflets of the plasma membrane.

D4H should also be helpful in elucidating the localization of cholesterol in the cytosolic leaflets of the cellular membranes during a variety of the cellular events such as phagocytosis, macropinocytosis, or autophagy. Furthermore, the dynamics of cholesterol in the cytosolic leaflet of the plasma membrane can be examined by fluorescence recovery after photobleaching (FRAP) and single particle tracking (SPT) analysis. It would be important to compare the dynamics of D4H labeled cholesterol with that of the Bodipy (or TopFluor) labeled cholesterol using the advanced microscopic techniques [85]. Cholesterol clusters in the cytosolic leaflets of the plasma membrane could be visualized by electron microscopy and super-resolution microscopic techniques [84,86]. Examination of the existence of cholesterol clusters in the cytosolic leaflet of the plasma membrane is potentially a very fruitful area of research. With regards to cholesterol in the cytosolic leaflet of other organelles, we have preliminarily found that mCherry-D4H localizes to endosomes and lipid droplets (unpublished data). Further characterization of the D4H positive endosomes and lipid droplets could provide novel functions of cholesterol during membrane trafficking by (i) co-expression of mCherry-D4H with other fluorophore-tagged organelle markers; or (ii) expression of mCherry-D4H in cells, in which organelles are stained with specific dyes.

## 7. Concluding Remarks

In this review, we highlight a variety of the cholesterol biosensors derived from PFO. Despite the usefulness of the PFO-derived cholesterol biosensors to visualize and detect intracellular cholesterol, attention should be paid to the fact that changes of binding of the PFO-derived cholesterol biosensors do not always directly correlate with changes in the cholesterol concentration in membranes. For instance, previous studies have shown that the binding of full-length PFO to cholesterol is affected by the surrounding environment in the membranes [19,64,87,88]. For example, full-length PFO bound better to cholesterol molecules that are loosely packed with unsaturated acyl chains species of phosphatidylcholine [19]. Consistent with this observation, sphingomyelin, which ensures cholesterol is well packed in the membranes, inhibited the binding of full-length PFO to cholesterol [87]. Additionally, when cholesterol is reconstituted with endoplasmic reticulum enriched phospholipids, a decrease in the threshold of cholesterol for binding by full-length PFO is observed [64,88]. Thus, changes in the distribution of cholesterol are detected using the PFO-derived cholesterol biosensors; investigators should always consider the possibility of other changes, especially changes in the phospholipids’ composition. The influence of surrounding phospholipids on the binding of the PFO-derived cholesterol biosensors to cholesterol can be easily examined through some *in vitro* binding assays (e.g., liposome sedimentation assay, Förster resonance energy transfer based assays).

Finally, it is also important to understand both the advantages and limitations of each PFO-derived biosensor. Selection of the specific biosensors will be critical for successful experiments. Particularly, a combination of PFO-derived cholesterol biosensors and high-end microscopic techniques (e.g., high-resolution electron microscopy, super-resolution microscopy, FRAP, SPT) would provide novel insights into the cellular properties of cholesterol, its interactions with other lipids, and its control by cellular proteins and trafficking pathways.

## Figures and Tables

**Table 1:** Cholesterol biosensors derived from PFO (Perfringolysin O).

Name	Structure	Mutations	Type	Temperature	Localization	Devices for Detection	Live-Imaging
PFO*	Full length	Y181A/C459A	^125^I-labeled recombinant proteins	4 °C	PM (exofacial)	Scintillation counter	No
BCθ	Full length	No	Biotinylated recombinant proteins	RT	PM, endosomes	Electron microscopy/Fluorescence microscopy	No/Yes (with Avidin)
D4	Domain 4	No	Fluorophore-labeled recombinant proteins	RT or 4 °C	PM (exofacial)	Fluorescence microscopy, PALM Flow cytometry	Yes
D4H	Domain 4	D434S	Vector-based (with fluorophore)	37 °C	PM, endosomes (cytosolic)	Fluorescence microscopy	Yes

PM: plasma membrane, RT: room temperature, PALM: photoactivated localization microscopy, *: unable to oligomerize.
